# Development of an awareness-based intervention to enhance quality of life in severe dementia: trial platform

**DOI:** 10.1186/1745-6215-11-73

**Published:** 2010-06-25

**Authors:** Linda Clare, Robert T Woods, Rhiannon Whitaker, Barbara A Wilson, Murna Downs

**Affiliations:** 1 School of Psychology, Bangor University, Gwynedd LL57 2AS, UK; 2 Dementia Services Development Centre, Bangor University, Gwynedd LL57 2AS, UK; 3 North Wales Organisation for Randomised Trials in Health and Social Care, Bangor University, Gwynedd LL57 2AS, UK; 4 MRC Cognition and Brain Sciences Unit, Cambridge, UK; 5 Bradford Dementia Group, Bradford University, Bradford, UK

## Abstract

**Background:**

Quality of residential care for people with severe dementia is in urgent need of improvement. One reason for this may be the assumption that people with severe dementia are unaware of what is happening to them. However, there is converging evidence to suggest that global assumptions of unawareness are inappropriate. This trial platform study aims to assist care staff in perceiving and responding to subtle signs of awareness and thus enhance their practice.

**Methods/Design:**

In Stage One, a measure of awareness in severe dementia will be developed. Two focus groups and an expert panel will contribute to item and scale development. In Stage Two observational data will be used to further develop the measure. Working in four care homes, we will recruit 40 individuals with severe dementia who have no, or very limited, verbal communication. Data on inter-rater reliability and frequency of all items and exploratory factor analysis will be used to identify items to be retained. Test-retest and inter-rater reliability for the new measure will be calculated. Correlations with scores for well-being and behaviour and with proxy ratings of quality of life will provide an indication of concurrent validity. In Stage Three the new measure will be used in a single blind cluster randomised trial. Eight care homes will participate, with 10 residents recruited in each giving a total sample of 80 people with severe dementia. Homes will be randomised to intervention or usual care conditions. In the intervention condition, staff will receive training in using the new measure and will undertake observations of designated residents. For residents with dementia, outcomes will be assessed in terms of change from baseline in scores for behaviour, well-being and quality of life. For care staff, outcomes will be assessed in terms of change from baseline in scores for attitudes, care practice, and well-being.

**Discussion:**

The results will inform the design of a larger-scale trial intended to provide definitive evidence about the benefits of increasing the sensitivity of care staff to signs of awareness in residents with severe dementia.

**Trial Registration:**

ISRCTN59507580 http://www.controlled-trials.com.

## Background

Quality of care for people with severe dementia in residential settings is poor and in urgent need of improvement [1]. This represents perhaps one of the major current challenges in dementia care [2]. In the UK, estimates of the number of people with dementia living in care homes range from a quarter of a million people to 368,400 [3,4], with two-thirds of these residents having a moderate to severe degree of dementia. In the US, half of all nursing home residents have moderate to severe dementia. The need for improvements in quality of care has been highlighted by the Department of Health's 'Dignity in Care' campaign and by the Joint Committee on Human Rights [5]. 'Dignity' is defined in this context as involving care that promotes the self-respect of the older person [6]; treating the person as an object rather than a person would be one example of the care practices this campaign is aiming to eradicate. Perhaps the greatest challenge for care homes is to uphold the dignity of people with severe dementia, who may need considerable help with basic functions, such as feeding, dressing, washing and toileting, and whose communication ability may be very limited. The current emphasis on dignity implies that the care of people who appear to lack self-awareness or awareness of their surroundings may be especially challenging for care staff. The presumed unawareness of the person with dementia may lead to marginalisation, where caregiving interactions become task-focused, pay limited attention to the person's reactions and fail to acknowledge the person's remaining ability to respond to stimulation. This study, funded by the Medical Research Council, will revisit and challenge these assumptions of unawareness, so as to provide a context for dignity in the care of people with severe dementia.

The capacity to show awareness can be defined as the ability to make an accurate appraisal or appropriate response in a given domain, for example in relation to internal states, external stimuli, symptoms, or changes in functioning. Awareness may be expressed at various levels, from basic perceptual and sensory awareness through to sophisticated self-awareness [7]. Individual variability in aspects of awareness and in relation to different domains has been documented in people with mild to moderate dementia, with studies focusing mainly on identification of deficits in awareness. Neuropsychological models have attempted to account for this variability in terms of changes in cognitive functioning [8] while others have pointed to the role of psychological factors in determining response to illness [9]. In recent comprehensive reviews we have drawn these perspectives together in a biopsychosocial framework [10,11].

Limited attention has been given to awareness and subjective experience in people with dementia living in residential care, who are likely to have moderate to severe levels of impairment. It has been suggested that awareness lessens as scores on cognitive tests decline [12], with the implication that at a certain stage of cognitive decline awareness is lost. However, correlational studies focusing mainly on the earlier stages of dementia suggest that the relationship between level of cognitive decline and degree of unawareness is not straightforward [13]. Therefore, some awareness about one's situation and functioning might be retained even in the presence of severe cognitive impairment [14]. Our comprehensive review of the literature reporting assessment of awareness in dementia [15] found only two small-scale studies focused on awareness in people with severe dementia. However, we have recently completed what we believe is the first systematic study of awareness in people with moderate to severe dementia [16]. This involved analysis of 304 interviews with 80 individuals with dementia residing in long-term care. MMSE scores ranged from 0-20 with a modal score of 0, indicative of severe cognitive impairment. All participants showed awareness in a range of domains, while about two-thirds also showed unawareness in at least one area. Our findings provided evidence that people with moderate to severe dementia are often aware to a considerable degree, and perhaps more than is often assumed by those most closely involved in providing care. Focus groups held with care staff suggested that where residents talk in ways that suggest an unrealistic appraisal of their situation or a confusion between past and present reality, they are likely to be regarded as globally 'unaware', negating the aspects of retained awareness that are also evident. While this study provided evidence about awareness in the context of retained capacity to communicate verbally, those for whom verbal communication was very limited or absent were not included.

For individuals who cannot communicate verbally, level of awareness can only be assessed through detailed observation of their behaviour and reactions. Here we can draw parallels with research on other groups where verbal communication is compromised. Following severe traumatic brain injury, demonstrable awareness is absent in coma and vegetative states, but people in minimally conscious states show subtle but definite behavioural indications of awareness with evidence of meaningful interaction with the environment [17]. The development of a comprehensive assessment measure based on detailed observation of behaviour, the Wessex Head Injury Matrix (WHIM) [18], has indicated that such patients often have a greater degree of awareness than previously assumed. The WHIM, which has good reliability and validity, has been found to be a more appropriate and sensitive measure than other tools currently used to assess emergence from coma and the minimally conscious state [19]. The WHIM has been used to demonstrate the effects of interventions such as postural change on levels of awareness and responsiveness for people in the minimally conscious state, supporting the view that some kinds of sensory stimulation may promote the expression of awareness [20].

In view of this, it is likely that people with severe dementia who do not communicate verbally may show awareness, at least at the sensory and perceptual level, and may express through their behaviour a response to environmental stimuli and interactions with caregivers. Consistent with this, studies have demonstrated preserved reactions to sensory stimuli in people with very severe dementia [21]. Observation of reactions and responses to particular stimuli will provide an indication of the person's awareness at any given time. If care staff can be trained to observe these behavioural indications of awareness, and understand them as such, this should help to increase their sensitivity to the needs of the person with severe dementia and to find ways of increasing awareness and responsiveness through, for example, sensory stimulation, postural change, or environmental adaptation, with benefits to residents in terms of quality of life and quality of care, and to staff in terms of increased job satisfaction and morale. Recent parallel accounts from a practitioner and a caregiver suggest that working in this way can increase sensitivity to responses and needs, and enrich the caregiving experience [22,23].

An approach of this kind requires the ability to influence the attitudes and practices of care staff. Training must be carefully designed to achieve the required outcomes. A systematic review of staff training in dementia care in nursing homes [24] identified 21 studies, mainly from the USA. Results in terms of outcomes for staff were generally positive, but outcomes for residents appeared to be better, and maintained longer, where the training involved a combination of initial information designed to enhance knowledge, attitudes and beliefs, environmental change to support the implementation of new skills, and the provision of cues or reminders to prompt and reinforce the use of new skills. Of particular relevance to the current proposal, providing care staff with 10 hours of training in being sensitive to non-verbal signals of emotion in people with severe dementia [25] resulted in significant increases in positive affect 6 weeks after the training for the residents in the intervention group, but this was not maintained over time. In our study we intend to address issues of maintenance by giving staff an instrument they can use routinely to assist them in focusing in detail on the person's level of awareness and emotional sensitivity. In summary, training needs to consist of more than just teaching sessions; our proposal is that by regular use of the instrument, staff will be able to operationalise indicators of awareness, and learn to apply them to the individual. This in turn will help staff to have more understanding of and empathy with residents with severe dementia; greater empathy and more positive attitudes are associated with reduced risk of staff burnout and increased job satisfaction [26].

In order to enable staff to undertake effective and systematic observation of behaviour, a reliable instrument for the assessment of awareness in severe dementia is required. This instrument should be simple to use and easy to integrate within the tasks of caregiving, and should provide clear operational definitions of the behaviours to be monitored. Existing measures do not meet this requirement. Dementia Care Mapping [27] has been useful in evaluating quality of care in residential settings, but focuses on more complex behaviours and requires an extensive time commitment from specially-trained and accredited raters. DCM lacks sufficient sensitivity to capture subtle aspects of behaviour in people with severe dementia [28]. Dementia Care Mapping (DCM) was designed as a standalone practice development tool, and was not intended for use as part of everyday practice. While it provides clear operational definitions of more complex behaviours, it was not designed to capture subtle aspects of behaviour in people with severe dementia. Other measures, such as the Positive Response Scale [28] or the Quality of Life in Late-Stage Dementia scale [29], rely to a considerable extent on interpretation of responses by the rater (for example, whether the residents shows 'enjoyment'), rather than recording discrete observable behaviours (for example, whether the resident tracks the carer with his/her eyes for at least 30 seconds). Fortunately, a valuable model is provided by the WHIM, described above, which offers a simple but reliable format for members of the multi-disciplinary team, as they go about their duties, to observe and record discrete behaviours exhibited by patients with severe brain injury. We propose to develop and validate a new measure for the assessment of awareness in people with severe dementia, based on the content and format of the WHIM. This will help staff to identify behavioural expressions of awareness in a way that can be readily integrated with on-going care provision. We will then conduct an initial intervention study in the form of a cluster randomised trial (CRT) in which we will train staff to use the measure with residents who have severe dementia, support them in adapting their practice to take account of their observations, and evaluate the impact on quality of care and on residents' quality of life. This will inform the design of a larger-scale trial, for which our core hypothesis is that training care staff to identify indications of awareness in people with severe dementia and enhance their care practice on the basis of their observations will result in improved quality of care and quality of life for the residents concerned.

## Design and Methods

This trial platform study will be conducted in three stages. First, we will explore the perspectives of paid and family carers, so as to incorporate their diverse experience and expertise, and with assistance from an expert panel we will prepare an initial draft of the proposed instrument. Second, we will conduct an observational study as a basis for further developing the new measure and evaluating its reliability and validity. Exploratory factor analysis will assist in refining the tool and hypothesising its factor structure. Third, we will conduct a randomized controlled trial (RCT) of an intervention in which staff are trained to use the new measure and outcomes are evaluated. Each stage is outlined in more detail below.

## Ethics and research governance

Establishing a sound ethical framework for this research is a key priority, as it involves observation of people with dementia, a vulnerable group who may not be able to give informed consent. The provisions of the Mental Capacity Act (2005) will be followed. In each case, the research will be explained to the potential participant in a conversational manner, at a time and in a place where the person is most likely to feel relaxed, with a supporter present where available. Consent will be sought where the person appears to understand and retain the information given, and makes a clear expression of their decision. Realistically, as the project will focus on people with severe dementia, we anticipate that most, if not all, participants will be unable to give informed consent. In each case where the person is unable to give informed consent, a suitable person will be approached to give an opinion on the person's likely views regarding participation. Wherever possible this will be a family member; where no family member is available, a suitable person will be asked to be the consultee, following appropriate guidance from the National Assembly for Wales. This research can only be effective if it includes people with severe dementia; the concern regarding dignity and human rights of such individuals in care settings makes this an important area to address, with the potential for benefits for this population. The research carries minimal risks for participants. All observational work will be undertaken in public areas of the homes, and on each occasion the researchers will explain the purpose of their presence in general terms to residents in the area. For individual assessments, the researchers will be trained to identify non-verbal indications that the person is unwilling to be involved in the research, and will not continue with any procedure which the person finds distressing. With regard to research governance, the project will be sponsored by Bangor University. A favourable ethical opinion has been granted by the relevant University ethics committee and by the North West Wales NHS Research Ethics Committee (reference 08/WNo01/60).

## User involvement

We will work closely with the User Carer Programme of NEURODEM Cymru (hosted by the Alzheimer's Society in Wales) to ensure that the perspectives of family carers and advocates for people with dementia contribute to the development of the project. A project steering group will be established and representatives from the group will meet regularly with the research team. Results will be disseminated to lay groups through links with the Alzheimer's Society, and through the Residents & Relatives Association.

## Stage One. Item and scale development

### Design

This is a qualitative study using focus group methods and an expert panel.

### Participants

Focus group participants will include managers, nurses, care staff and family caregivers recruited through participating care homes and local Alzheimer's Society branches.

### Procedure

Two focus groups will be conducted, one with formal carers including care home managers, nursing staff and care assistants (n = 10), and one involving family carers with relatives in care homes or in the community (n = 10). Participants will discuss their views on awareness, identify what they consider key indicators of awareness in people with severe dementia, and consider the relevance, appropriateness and applicability of the WHIM items and format. An expert panel consisting of psychometricians and practitioners and researchers in dementia care (n = 10), recruited through professional networks with which the applicants are associated (e.g. DeNDRoN), will convene to assimilate the findings of the focus groups, suggest item reduction and modification of the WHIM, and formulate an appropriate rating system.

### Analysis

Qualitative thematic and content analyses of transcripts of the focus group sessions will be conducted.

### Outputs

A preliminary version of the new measure will be prepared.

## Stage Two. Development of the awareness measure

### Design

Observational data will be used alongside ratings made by care staff and the research team in order to further develop the measure of awareness in people with severe dementia. Data will be subjected to exploratory factor analysis to further refine the tool for use in Stage Three.

### Participants

We will identify 4 care homes in North Wales, within which we will recruit 40 individuals with severe dementia who have no, or only very limited, verbal communication.

### Measures for assessment of the person with dementia

• Severity of dementia: Functional Assessment Staging (FAST) [30], a widely-used brief system for categorising severity of impairment in people with dementia. [In the initial funding application we proposed to use the Global Deterioration Scale [31], but following initial piloting we decided to replace it with the FAST which provides a more fine-grained classification.]

• Cognitive functioning: Guy's Advanced Dementia Schedule [32]; a structured assessment of cognitive ability that involves measuring responses to familiar objects. [In the initial funding application we proposed to use the Severe Impairment Battery [33], but as initial piloting indicated floor effects on this measure we replaced it with the GADS.]

• Behaviour: Behavioural Assessment Scale of Later Life (BASOLL) [34]; a reliable and valid rating of self-care ability, functioning and behaviour, completed by staff.

• Well-being: Positive Response Scale (PRS) [28]; an observational scale completed by a trained rater that focuses on the person's affective response to the environment.

• Quality of life: Quality of Life in Late-stage Dementia (QUAL-ID) [29]; an 11-item scale completed by a proxy with reference to the person's quality of life in the preceding week.

• Awareness: Wessex Head Injury Matrix (WHIM) [18,19]; an hierarchical list of 62 observable behaviours, ranging from 'looks at person briefly' to 'knows the name of one staff member'. This will be modified in line with recommendations from Stage One.

### Procedure

All participants will be observed for two 30-minute periods using the PRS and assessed with the GADS to provide a profile of their current level of well-being and cognitive functioning. A designated member of the care staff will be interviewed for each participant, and will provide background information, make ratings of severity of dementia (FAST) and behavior (BASOLL), and provide proxy ratings of quality of life (QUAL-ID). Where possible a family member will be interviewed to obtain background information and provide parallel proxy ratings of quality of life. Next, observational data using the modified WHIM will be collected for all participants. Observations of each participant will be carried out by a single rater in public areas of the care home, while participants are awake, over a period of 30 minutes on 5 different occasions and at different times of the day in order to capture any fluctuations in levels of awareness. A second independent rater will concurrently observe a sample of 12 residents (3 from each home) on all 5 occasions. Any further additional behaviours identified during the observations will be operationally defined by the research team in concise and concrete terms. Draft definitions will be tested prospectively with participants and further revised if necessary.

### Analysis

(a) Determining the content of the new measure: Test-retest reliability for *each individual item *will be calculated using data from all 40 participants relating to two assessment sessions, held at the same time of day, one week apart. Inter-rater reliability for *all items *will be assessed using data from the 12 participants who were observed concurrently by two independent raters. Behaviours that cannot be rated reliably will be excluded, as will any behaviours that occurred in fewer than 5% of assessments or were not observed at all. Systematic exploratory factor analysis (EFA) will be used to generate an hypothesised factor structure and to further modify the tool by excluding non-informative items. Sample size estimates for an EFA range from 5 to 20 or more subjects required per item, depending on the nature of the data. Five assessments with each of 40 participants will yield 200 uses of the tool. We acknowledge that a dependency structure might exist and will test for this, but it is unlikely to reduce the effective sample size below 100, which will be sufficient provided the emergent scale consists of no more than 20 items.

(b) Determining the properties of the new measure: Test-retest and inter-rater reliability for the new measure will be calculated on the same basis as for the constituent items. The relationship between awareness and cognitive impairment will be assessed in terms of correlations between scores on the new measure and scores on the GADS. Higher levels of observed awareness are predicted to be associated with greater well-being and higher proxy ratings of quality of life; therefore, correlational analysis will be used to identify the relationship between scores on the new measure and scores for well-being (PRS), behaviour (BASOLL), and quality of life (QUAL-ID proxy scores produced by staff and family carers), providing an indication of concurrent validity.

### Outputs

The key output from this stage will be a reliable, valid and parsimonious measure of awareness in people with severe dementia that takes into account user perspectives, and which is presented in a format that can be easily used by care staff, accompanied by a comprehensive and accessible user manual.

## Stage Three: Intervention trial

### Design

**Figure 1:**
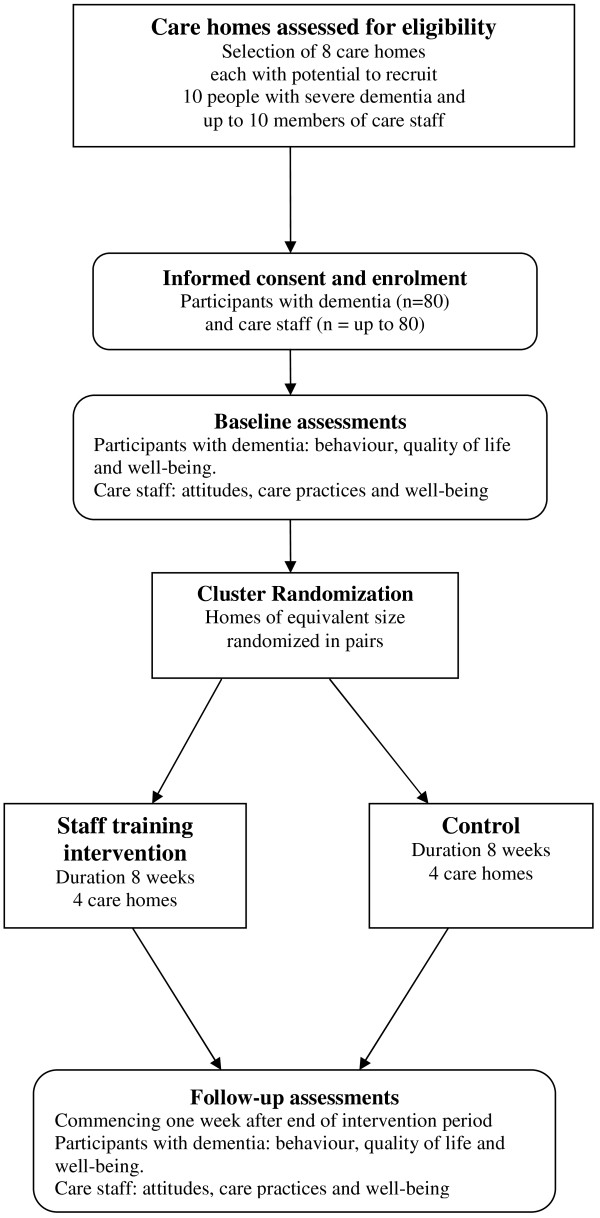
**CONSORT-style flowchart for Phase 3**.

In this CRT paid carers will be trained to use the new measure of awareness and the effects on resident well-being and quality of life will be evaluated. Eight care homes will be recruited to the trial. Half the homes will be randomised to receive the training intervention; the remainder will receive no additional input. Baseline assessments will be completed in pairs of homes of similar size and type (public or independent sector) before one of the pair is randomised to receive the intervention. A CONSORT-style flowchart is shown in Figure 1.

### Participants

Stage Three will be conducted with 8 care homes which were not involved in the previous stage. We will recruit 10 residents per home giving a total sample of 80 people with severe dementia who have no, or only very limited, verbal communication.

### Measures

(a) Measures for assessment of the person with dementia

• Awareness will be assessed using the new measure.

• Severity of dementia: Functional Assessment Staging (FAST) [30].

• Cognitive functioning: Guy's Advanced Dementia Schedule (GADS) [32].

• Behaviour: Behavioural assessment scale of later life (BASOLL) [34].

• Well-being: Positive Response Scale (PRS) [28].

• Quality of life: Quality of Life in Late-stage Dementia (QUAL-ID) [29].

(b) Measures for care staff

• Staff attitudes: Approaches to Dementia Questionnaire (ADQ) [35,36]; a 19-item reliable and valid scale assessing person-centred and hopeful attitudes to people with dementia.

• Care practice: The Dementia Care Practitioner's Assessment (DCPA) [37]; an observational schedule evaluating quality of care provided by staff.

• Well-being: Maslach Burnout Inventory (MBI) [38]; a brief self-report questionnaire providing scores for emotional exhaustion, depersonalisation and personal accomplishment.

• General Health Questionnaire (GHQ-12) [39]; a brief, well-validated measure of psychological distress.

### Intervention

In the intervention condition, care staff will be introduced to the measure in teaching sessions, gain hands-on experience of using the measure under the guidance of a trainer, and then undertake observations of allocated residents and receive group supervision to support the development of care practice with those residents. An accredited trainer will advise on, and support the process of, staff training. For each home, the intervention period will last for 8 weeks. In weeks 1 - 2, two 90-minute training sessions will be provided, and each staff member involved will be given an individualised observation schedule. In weeks 3 - 8, each staff member will conduct six 10-minute observations per week and receive 30 minutes face-to-face or telephone contact with the researcher. Observations will be scheduled so that (a) pairs of staff members make independent observations of the same resident; (b) each staff member observes each identified resident on a number of occasions; (c) each staff member observes at least three different residents; and (d) each staff member observes at least two residents over an extended period of at least three weeks. Observations will be conducted in public areas of the home while the residents are awake. All staff will participate in fortnightly one-hour group supervision sessions, where they will be encouraged to consider variations in residents' level of awareness, and to identify and implement ways of supporting increased levels of awareness.

### Procedure

Participants will be assessed initially with the GADS and observed for two 30-minute sessions with the PRS, a staff member will be interviewed to obtain background information and ratings on the FAST and BASOLL and proxy ratings on the QUAL-ID, and where possible a family member will be interviewed to obtain proxy ratings on the QUAL-ID. Staff members will also complete the ADQ, MBI and GHQ-12. Care practice will be observed for 6 hours in each home using the DCPA. For homes in the intervention condition, staff will participate in the training and observational work. For homes in the usual care condition, no staff training will be provided. After 8 weeks all measures used in the initial assessment will be repeated by a researcher blind to group allocation.

### Data analysis

The data analysis will have four purposes: first, to estimate the intra-class correlations (ICC) required to power a large-scale trial; second, to conduct a confirmatory factor analysis on the developed tool in order to test the hypothesised structure which emerged from stage 2; third, to develop and test an analysis plan based on multilevel modelling; and fourth, to examine change from baseline scores on all outcome measures by randomisation group to assess possible effect size associated with both intervention and control arms (the repeated observations and ratings made by staff may trigger some changes in the control homes). A sample size of 8 care homes with 10 participants per home achieves 90% power to detect an ICC of 0.3 under the alternative hypothesis when the ICC under the null hypothesis is 0.0 using an F-test with a significance level of 0.05 [40]. The proposed sample size will also yield estimates of the standardised effect size of the change from baseline in either arm of the trial with a 95% confidence interval of +/- 0.3.

### Outputs

The information obtained about the ICC and effect size will make it possible to determine the sample size required for a definitive, larger-scale trial. In addition, this initial RCT will supply additional information about the new measure, provide an opportunity to test the intervention in a systematic way, and allow us to gain a clear picture of the practicalities involved in implementing the research method in the care homes. Findings will be incorporated into existing training provision for care staff offered by the Bangor Dementia Services Development Centre and the Bradford Dementia Group.

## Discussion

There is evidence to suggest that awareness at the level of sensory and perceptual responses can be observed even in people with very severe or end-stage dementia, and that the extent to which this awareness can be expressed or identified is influenced by the context and the nature of the caregiving interactions involved [41]. This study will provide an accessible, user-friendly tool that care staff can use to structure their observations of residents' awareness in the course of their daily work. Care staff participating in the intervention in Stage 3 of the study will be given the opportunity to focus on, observe, and discuss the way in which residents with severe dementia can demonstrate awareness, using this tool. The experience they gain is expected to assist them in providing sensitive, high-quality care, with beneficial effects on the quality of life of residents. Stage 3 of the study will offer preliminary evidence regarding this hypothesis. If the hypothesis is supported, our aim will be to develop a large-scale, multi-site RCT in order to provide definitive evidence about the benefits for care staff, residents and organizations of increasing the sensitivity of care staff to signs of awareness in residents with severe dementia. While such a trial is likely to present considerable methodological and practical challenges, the development of evidence-based approaches to improving quality of care and quality of life for residents with severe dementia represents an important priority.

## Competing interests

The authors declare that they have no competing interests.

## Authors' contributions

LC conceived of the study, contributed to study design, and took the lead in preparing the protocol. RTW and RW contributed to study design and preparation of the protocol. BAW and MD contributed to study design and reviewed the protocol. All authors read and approved the final manuscript.
